# Interface-reinforced high-capacity fiber cathode for wearable Li–S batteries

**DOI:** 10.1093/nsr/nwae262

**Published:** 2024-07-30

**Authors:** Lei Huang, Tianzhu Zhou, Siyu Zhu, Tianqi Yang, Xuhui Zhou, Bing He, Shuai Wang, Wei Yan, Lei Wei

**Affiliations:** School of Electrical and Electronic Engineering, Nanyang Technological University, Singapore 639798, Singapore; School of Electrical and Electronic Engineering, Nanyang Technological University, Singapore 639798, Singapore; School of Materials Science and Engineering, Nanyang Technological University, Singapore 639798, Singapore; Department of Physics, City University of Hong Kong, Hong Kong 999077, China; School of Electrical and Electronic Engineering, Nanyang Technological University, Singapore 639798, Singapore; School of Electrical and Electronic Engineering, Nanyang Technological University, Singapore 639798, Singapore; School of Electrical and Electronic Engineering, Nanyang Technological University, Singapore 639798, Singapore; State Key Laboratory for Modification of Chemical Fibers and Polymer Materials, College of Materials Science and Engineering, Donghua University, Shanghai 201620, China; School of Electrical and Electronic Engineering, Nanyang Technological University, Singapore 639798, Singapore

**Keywords:** Li–S batteries, flexible electrodes, interface interactions, fiber-shaped devices, energy storage

## Abstract

Fiber-shaped Li–S batteries are attractive for constructing smart textiles as flexible power solutions due to their high theoretical specific capacity, flexibility and wearability. However, severe interfacial issues, such as the shuttle effect of polysulfides on the cathode side, lead to capacity decay and poor lifespan of the batteries. Herein, we report a fiber-shaped composite cathode with collaborative interface interactions to maintain electrode integrity and boost electrochemical performance. In this architecture, nanosulfur-polyvinylpyrrolidone (nanoS-PVP) particles are uniformly implanted into the few-layer Ti_3_C_2_T*_x_* with outstanding electrical conductivity and then coated on aluminum (Al) fiber current collectors. Impressively, nanoS and soluble polysulfides are restricted to the cathode side via synergy physical confinement and chemical adsorption of Ti_3_C_2_T*_x_*. The PVP chains on the surface of the nanoS prevent the sulfur from agglomeration and bridge the Ti_3_C_2_T*_x_* by abundant hydrogen bonds. The enhanced interface endows the cathode with excellent mechanical flexibility, good adsorption of polysulfides and fast reaction kinetics. Consequently, the prepared Ti_3_C_2_T*_x_*/nanoS-PVP@Al cathode exhibits excellent cycling performance (capacity retention of 92.8% after 1000 cycles at 1 C), high-rate capacity (556.2 mAh g^−1^ at 2.0 C) and high linear capacity (22.9 mAh m^−1^). Additionally, the fiber-shaped Li–S battery works effectively under deformation and high/low-temperature conditions. It can be integrated into the fabric to power light emitting diodes or charge a smartphone wirelessly.

## Teaser

This work reports a fiber-shaped composite cathode with collaborative interface interactions to maintain electrode integrity and boost electrochemical performance for wearable Li–S batteries.

## INTRODUCTION

Future smart electronic textiles, capable of communicating, sensing, displaying and supplying electricity, have driven research and development in recent years [1,2]. Integrating energy-storage devices into textiles offers exciting opportunities for smart electronic textiles, poised to be self-powered without external power-supply equipment [3]. Conventional planar energy devices exhibit limited flexibility due to their bulky and rigid structures [4]. Consequently, fiber-shaped energy-storage devices are emerging to meet an ideal paradigm for energy-storage textiles that are soft, flexible, deformable and durable [5]. Compared with planar structures, the fiber shape endows them with promising advantages such as flexibility, light weight and feasibility for integration into large-scale textile systems [6,7]. However, these fiber-shaped devices face challenges primarily due to their limited energy-storage capacities [8]. Energy storage of conventional cathode materials such as lithium iron phosphate (LFP), lithium cobalt oxide and lithium nickel cobalt manganese oxide is intrinsically limited by the theoretical ceilings, implying the difficulty in meeting the energy supply needs of power-consuming wearable devices [9,10]. To seek capacity breakthroughs, researchers are exploring next-generation flexible batteries with high theoretical specific capacity and low cost, contributing to the fast growth of the wearable electronics market [11]. For instance, lithium–sulfur (Li–S) batteries, known for their exceptional theoretical specific capacity (1675 mAh g^−1^) and energy density (2600 Wh kg^−1^), represent a promising alternative [12–15]. Nevertheless, attempts to develop flexible sulfur cathodes face severe interfacial issues, such as the polysulfide shuttle effect and the formation of lithium dendrites, which result in capacity decay and poor lifespan of the batteries [16,17].

Several improvements have been made to develop eligible flexible sulfur cathodes to improve the geometric restrictions of planar devices. Fibrous materials are typically considered to be ideal hosts for sulfur due to their large surface area, excellent flexibility, good mechanical properties and lightweight characteristics. In particular, designing 1D Li–S fiber batteries can achieve maximum flexibility and wearability, which is beneficial for their successful application as power accessories in wearable devices. Previously, Peng *et al.* reported a 1D cable-shaped Li–S battery assembled with a sulfur-containing carbon nanostructured hybrid fiber cathode and Li wire [8]. The cable-shaped battery delivered an initial discharge capacity of 1051 mAh g^−1^ at 0.1 C and the capacity remained at ∼600 mAh g^−1^ after 100 cycles. Subsequently, two more fibrous Li–S fiber batteries with carbon-based hosts were developed, improving the electrochemical performance by constructing a composite carbon/sulfur cathode using wet spinning technology [18,19]. Except for carbon-based hosts, Liu *et al.* produced fibrous sulfur composite cathodes using stainless steel fibers as supports and current collectors, while the fiber battery exhibited a fast capacity decay compared with coin cells [20]. Despite several explorations, interface issues of fiber Li–S batteries have rarely been effectively addressed, especially in inhibiting the shuttle of polysulfides, which is crucial for improving the performance of Li–S batteries.

In this work, we present the construction of composite fiber sulfur cathodes with significant interface interactions, in which nanosized sulfur-polyvinylpyrrolidone (nanoS-PVP, ∼500 nm) are uniformly implanted into the few-layer Ti_3_C_2_T*_x_* and then uniformly coated on Al fiber current collectors. Note that nanoS and the intermediate soluble polysulfides can synergistically be hosted by Ti_3_C_2_T*_x_*, manifested as a synergistic effect of physical confinement and chemical adsorption. Meanwhile, the PVP chains on the surface of nanoS not only prevent the sulfur from agglomeration, but also form hydrogen bonding (H-bond) with surface groups (e.g. –OH) of Ti_3_C_2_T*_x_*, effectively suppressing sulfur detachment and maintaining cathode integrity. Density functional theory (DFT) calculations further demonstrate the strong interfacial interaction of lithium polysulfides with Ti_3_C_2_T*_x_*, as well as enhanced reaction kinetics. As a result, our prepared Ti_3_C_2_T*_x_*/nanoS-PVP@Al cathodes exhibit noteworthy flexibility, competitive electrochemical cycling performance, good high-rate capacity and high linear capacity. Moreover, fiber Li–S batteries work efficiently under deformation and high/low-temperature conditions, and can be integrated into commercial textiles to power light emitting diodes (LEDs) or woven into a phone bag for wirelessly charging a smartphone. Our work provides a general strategy to design advanced flexible cathodes for energy storage and conversion.

## RESULTS AND DISCUSSION

### The design strategy of composite fiber cathode

**Figure 1.**
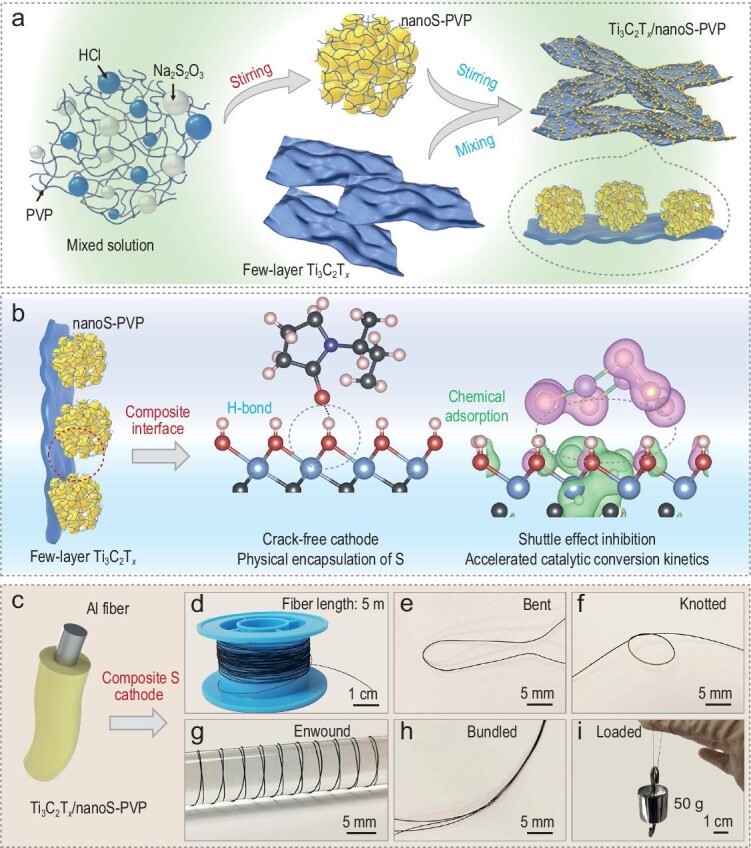
(a) Schematic fabrication process of Ti_3_C_2_T*_x_*/nanoS-PVP. (b) Schematic illustration of composite interfaces between nanoS-PVP and Ti_3_C_2_T*_x_*. (c) Structural diagram of fiber Ti_3_C_2_T*_x_*/nanoS-PVP@Al cathode. Photos of Ti_3_C_2_T*_x_*/nanoS-PVP@Al fibers: (d) collected on a spindle, (e) bent, (f) knotted, (g) enwound, (h) bundled and (i) loaded.

The two-step preparation of the Ti_3_C_2_T*_x_*/nanoS-PVP material is shown in Fig. 1a. First, the synthesis of nanoS-PVP was achieved through chemical reactions between Na_2_S_2_O_3_ and HCl, with PVP acting as nucleation sites and dispersants, effectively controlling the average size of the sulfur at the nanoscale. Due to the poor conductivity of sulfur, reducing its size was considered an effective way to improve its utilization [21]. Next, few-layer Ti_3_C_2_T*_x_* solutions (5 g L^−1^) were mixed with the collected nanoS-PVP and stirred, resulting in the formation of Ti_3_C_2_T*_x_*/nanoS-PVP composites due to group interactions on the surfaces of the nanoS-PVP and Ti_3_C_2_T*_x_*. Insight into the composite interfaces between nanoS-PVP and Ti_3_C_2_T*_x_* is proposed in Fig. 1b, highlighting its potential as an ideal cathode material for Li–S batteries: (i) nanoS-PVP particles are encapsulated by few-layer Ti_3_C_2_T*_x_* materials, improving the conductivity of the cathode and the utilization of active materials. (ii) Interface interaction ensures cathode integrity. The H-bond interaction between PVP chains wrapped in nanoS and Ti_3_C_2_T*_x_* prevents the active sulfur from detaching during cathode deformation, reducing the production of ‘dead S’. (iii) Shuttle effect inhibition—the synergistic effect of physical confinement and chemical adsorption (Ti_3_C_2_T*_x_*-polysulfides) suppresses the diffusion of polysulfides and reduces side reactions. (iv) The significant catalytic conversion ability of Ti_3_C_2_T*_x_*, reported in several battery systems, accelerates the catalytic conversion kinetics of polysulfides [22,23]. As a demonstration, fibrous Ti_3_C_2_T*_x_*/nanoS-PVP-based composite S cathodes were prepared for fiber-shaped Li–S batteries. Figure 1c illustrates the construction of the composite S cathode. In this architecture, Al fiber is applied as the current collector and Ti_3_C_2_T*_x_*/nanoS-PVP slurries are uniformly coated via the dip-coating method. A Ti_3_C_2_T*_x_*/nanoS-PVP@Al cathode with a length of 5 m can be easily fabricated (Fig. 1d), revealing the potential of scalable production. Furthermore, the abundant interface interactions endow fibers with a robust flexible structure that withstands rigorous deformations (e.g. bent, knotted, enwound and bundled, Fig. 1e–h) and an excellent tensile strength of ∼66.7 MPa (Fig. 1i and Fig. S1).

### Characterizations of fiber Li–S batteries

**Figure 2.**
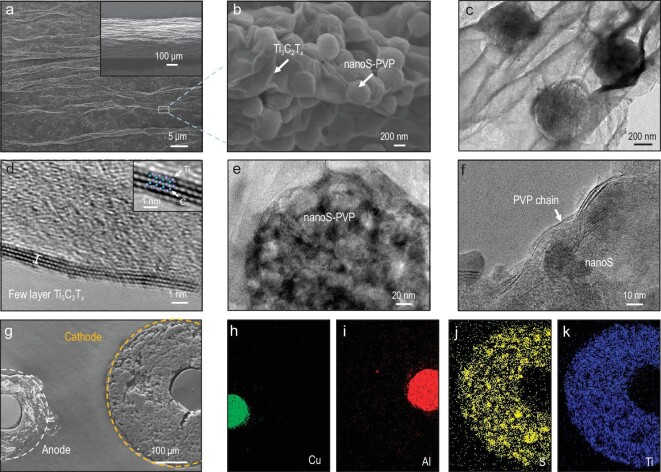
(a and b) Scanning electron microscopy (SEM) and (c) transmission electron microscopy (TEM) images of Ti_3_C_2_T*_x_*/nanoS-PVP-based fiber cathode. High-resolution TEM (HRTEM) images of (d) few-layer Ti_3_C_2_T*_x_*, (e and f) nanoS-PVP. (g) Cross-sectional SEM image and (h–k) corresponding energy-dispersive X-ray spectroscopy (EDS) elemental mappings of a Li–S fiber battery.

The morphology and microstructure of the prepared Ti_3_C_2_T*_x_*/nanoS-PVP@Al fiber cathode were investigated using scanning electron microscopy (SEM), transmission electron microscopy (TEM) and high-solution transmission electron microscopy (HRTEM). The average diameter of the Ti_3_C_2_T*_x_*/nanoS-PVP@Al fibers is ∼360 μm, featuring a slightly wrinkled and crack-free surface (Fig. 2a), which is superior to the carbon/nanoS-PVP@Al cathode that shows obvious cracks (Fig. S2). The enlarged view of Ti_3_C_2_T*_x_*/nanoS-PVP reveals the accurate distribution of nanoS-PVP and Ti_3_C_2_T*_x_* (Fig. 2b). The nanoS-PVP particles (∼500 nm) are well encapsulated by the few-layer Ti_3_C_2_T*_x_* (Fig. S3a), providing more effective dispersion compared with pure nanoS-PVP (Fig. S3b). Moreover, a continuous electron-transfer network is formed, improving the overall conductivity of the cathode and enhancing sulfur utilization efficiency. The constructed Ti_3_C_2_T*_x_*/nanoS-PVP@Al fibers show a high conductivity of ∼8.2 S cm^−1^, verifying good electron transfer of the Ti_3_C_2_T*_x_*/nanoS-PVP@Al electrode (Fig. S4). TEM characterization further confirms the microstructure of Ti_3_C_2_T*_x_*/nanoS-PVP (Fig. 2c). HRTEM images of Ti_3_C_2_T*_x_* (Fig. 2d) show a few-layer structure with a monolayer thickness (without residues) of ∼0.98 nm, consistently with previous reports [24]. As shown in Fig. 2e, nanoS-PVP displays a typical amorphous configuration. PVP chains are captured on the surface of nanoS (Fig. 2f) to anchor and stabilize nanoS on Ti_3_C_2_T*_x_*. A Cu/Li anode and gel electrolyte were also prepared to assemble full Li–S fiber batteries. As shown in Fig. S5, the Cu/Li fiber anode was fabricated by coating molten Li on the surface of Cu fiber (∼100 μm). After loading Li, the diameter of the composite Cu/Li fiber increases to 350 μm. The gel electrolyte was prepared by dissolving polyvinylidene fluoride-hexafluoropropylene (PVDF-HFP) into the commercial Li–S liquid electrolyte at 80°C and then cooling it down (Fig. S6). The cross-sectional SEM image (Fig. 2g) and the corresponding energy-dispersive X-ray spectroscopy (EDS) elemental mapping (Fig. 2h–k) demonstrate the integrity of the architecture and uniform element dispersion of electrode fibers.

**Figure 3.**
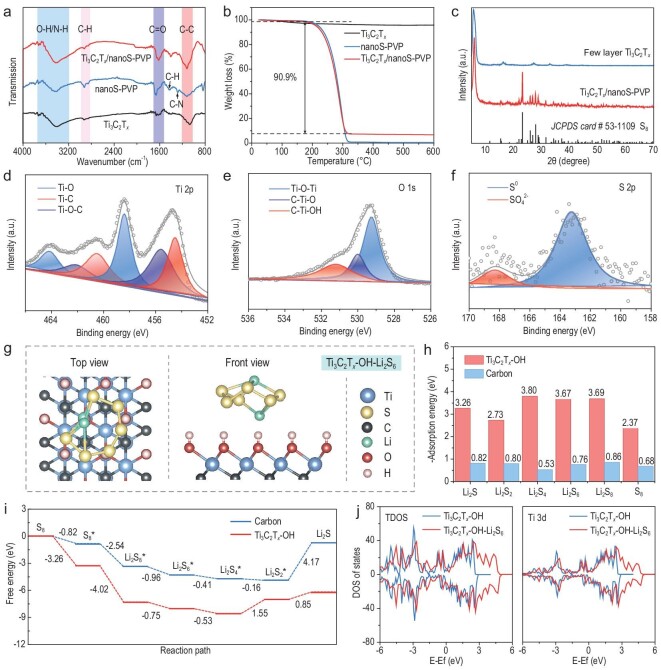
(a) Fourier transform infrared spectrometer (FTIR) spectra and (b) thermogravimetric (TG) analysis of Ti_3_C_2_T*_x_*/nanoS-PVP, nanoS-PVP and Ti_3_C_2_T*_x_*. (c) X-ray diffraction (XRD) patterns of Ti_3_C_2_T*_x_*/nanoS-PVP and few-layer Ti_3_C_2_T*_x_*. High-resolution X-ray photoelectron spectroscopy (XPS) spectra of (d) Ti 2p, (e) O 1s and (f) S 2p of Ti_3_C_2_T*_x_*/nanoS-PVP. (g) Top and front view of Li_2_S_6_ adsorbed on Ti_3_C_2_T*_x_*–OH. (h) Adsorption energies for sulfur species on Ti_3_C_2_T*_x_*–OH and pure carbon. (i) Gibbs free energy plots of S_8_-to-Li_2_S conversion on Ti_3_C_2_T*_x_*–OH and pure carbon. (j) Electronic density of states (DOS) analysis before and after Ti_3_C_2_T*_x_*–OH and Li_2_S_6_ interaction.

Fourier transform infrared spectrometer (FTIR) measurement further demonstrates the interfacial interaction between nanoS-PVP and Ti_3_C_2_T*_x_* (Fig. 3a). Ti_3_C_2_T*_x_*/nanoS-PVP composites exhibit characteristic peaks of nanoS-PVP and Ti_3_C_2_T*_x_* without new chemical bond formation. The C–N stretching vibration (∼1281.8 cm^−1^) and C=O vibration (∼1662.0 cm^−1^) of nanoS-PVP are weakened and have a certain shift (∼1278.2 cm^−1^ of C–N and ∼1619.4 cm^−1^ of C=O) after being combined with Ti_3_C_2_T*_x_*, indicating the H-bond interaction of C=O, C–N with Ti_3_C_2_T*_x_* residues (e.g. –OH) [25]. Thermogravimetric (TG) analysis (Fig. 3b) of Ti_3_C_2_T*_x_*/nanoS-PVP reveals the content of nanoS-PVP to be as high as 90.9 wt%. Considering the mass ratio of PVP to nanoS, it can be inferred that the sulfur loading of the composite is ∼81.8 wt%. Additionally, X-ray diffraction (XRD) and X-ray photoelectron spectroscopy (XPS) measurements were conducted to investigate the chemical compositions of the samples. As shown in Fig. 3c, the XRD pattern of Ti_3_C_2_T*_x_*/nanoS-PVP exhibits characteristic sulfur peaks (JCPDS 53–1109) and a (002) peak of few-layer Ti_3_C_2_T*_x_* (characterizations of multi-layer Ti_3_C_2_T*_x_* are supported in Fig. S7) [26], verifying the successful incorporation of sulfur on few-layer Ti_3_C_2_T*_x_*. As depicted in high-resolution Ti 2p spectra (Fig. 3d), the binding energies at 454.5/460.5, 455.6/462.1 and 458.4/464.2 eV belong to Ti–C, Ti–O–C and Ti–O, respectively [27]. The peaks of O 1s at 529.3, 530.0 and 531.2 eV correspond to Ti–O, C–Ti–O and C–Ti–OH, respectively (Fig. 3e) [28]. In the S 2p spectrum, two peaks at 163.2 and 168.3 eV are consistent with S^0^ and a small amount of sulfate (Fig. 3f) [29,30]. The above results indicate that the nanoS-PVP is bridged with few-layer Ti_3_C_2_T*_x_* to fabricate Ti_3_C_2_T*_x_*/nanoS-PVP through the H-bond.

### Determination of adsorption and catalytic conversion of polysulfides

To illustrate the strong interfacial interactions between Ti_3_C_2_T*_x_* and various sulfur species (Li_2_S_8_, Li_2_S_6_, Li_2_S_4_, Li_2_S_2_, Li_2_S and S_8_), and to simulate the immobilization and catalytic conversion kinetics, DFT calculations were conducted. In our case, Ti_3_C_2_T*_x_*–OH, of which the content is abundant through FTIR and XPS analysis, is employed as the modeling molecule to calculate the binding energies (ΔE) with different states of sulfur species. Figure 3g displays the top and front views of Li_2_S_6_ adsorbed on the Ti_3_C_2_T*_x_*–OH. Other adsorption structures of Ti_3_C_2_T*_x_*–OH and carbon absorbed with lithium polysulfides are exhibited in Figs S8 and S9. The summary bar chart of adsorption energies (Fig. 3h) shows that the ΔE of Li_2_S, Li_2_S_2_, Li_2_S_4_, Li_2_S_6_, Li_2_S_8_ and S_8_ with Ti_3_C_2_T*_x_*–OH is −3.26, −2.73, −3.80, −3.67, −3.69 and −2.37 eV, respectively, which is significantly higher than the ΔE absorbed with pure carbon surfaces (−0.82, −0.80, −0.53, −0.76, −0.86 and −0.68 eV). This demonstrates that the Ti_3_C_2_T*_x_*–OH possesses a much stronger affinity to the sulfur species, thereby enhancing its adsorption capacity for soluble lithium polysulfides and improving electrochemical performance. The enhanced adsorption capabilities of Ti_3_C_2_T*_x_* are further verified by *ex situ* visible adsorption measurements and ultravioletvisible (UV–vis) spectroscopy (Fig. S10). The pure Li_2_S_6_ solution was applied as the representative for soluble long-chain polysulfide species [31]. Equal amounts of Ti_3_C_2_T*_x_* and carbon samples were immersed into the Li_2_S_6_ solution for 24 h and the corresponding optical images were recorded (Fig. S10a). After 24 h, the solution with the Ti_3_C_2_T*_x_* became clear and transparent, while the solution with carbon was still yellowish, indicating that Ti_3_C_2_T*_x_* has stronger polysulfide adsorption ability than carbon. UV–vis spectroscopy after adsorption further confirmed these results (Fig. S10b). The densities of characteristic Li_2_S_6_ adsorption peaks of Ti_3_C_2_T*_x_* and carbon samples both decrease. Especially for the Ti_3_C_2_T*_x_* sample, the characteristic peak has almost disappeared, suggesting that soluble polysulfides are adsorbed by Ti_3_C_2_T*_x_*.

Figure 3i illustrates that Ti_3_C_2_T*_x_*–OH has a lower Gibbs free energy than carbon during the reaction from S_8_ to Li_2_S, demonstrating easier reduction processes of polysulfides on the surface of Ti_3_C_2_T*_x_*–OH. Notably, the overall free energy on the surface of Ti_3_C_2_T*_x_*–OH and carbon is −6.16 and −0.72 eV, respectively, suggesting that the whole reaction process on the surface of Ti_3_C_2_T*_x_*–OH is more inclined towards being thermodynamically spontaneous [32]. In the rate-limiting steps of the reaction path with positive free energy, the Ti_3_C_2_T*_x_*–OH reduces the overall positive energy from 4.17 to 2.40 eV, indicating improved reaction kinetics for lower reaction barriers. Moreover, electronic density of state (DOS) analysis of the Ti_3_C_2_T*_x_*–OH on the redox and transformation of polysulfides was performed to reveal interfacial charge regulation mechanisms. The total DOS and the Ti 3d orbital projected DOS of Ti_3_C_2_T*_x_*–OH before and after Li_2_S_6_ probe molecule adsorption were investigated. Due to the chemical interaction between Ti_3_C_2_T*_x_*–OH and Li_2_S_6_, the DOS curves show a certain deviation and significant electron concentration enhancement peaks are observed near Ef. More charge compensation from the d-band of the Ti atom enters the adsorbed Li_2_S_6_, resulting in more d–p orbital hybridization and enhancing the charge compensation effect, thus promoting interfacial redox and conversion kinetics [33]. Furthermore, the accelerated polysulfide catalytic conversion ability of the Ti_3_C_2_T*_x_* host is validated via cyclic voltammogram (CV) curves of Li_2_S_6_ symmetric batteries (Fig. S11). Compared with the pure carbon sample, the Ti_3_C_2_T*_x_* assembled battery shows higher current density and sharper redox peak, verifying that the Ti_3_C_2_T*_x_* has better polysulfide catalytic conversion efficiency and faster reaction kinetics.

### Electrochemical performance of fiber Li–S batteries

**Figure 4.**
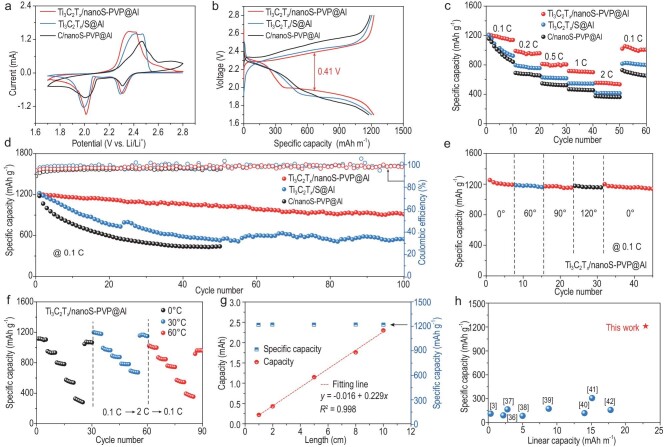
Electrochemical performances of Ti_3_C_2_T*_x_*/nanoS-PVP@Al, Ti_3_C_2_T*_x_*/S@Al and C/nanoS-PVP@Al fiber cathodes. (a) Cyclic voltammogram (CV) curves at a scan rate of 0.1 mV s^−1^, (b) galvanostatic charge/discharge plots, (c) rate performance, (d) cycling performance at 0.1 C. (e) The specific capacities of the fiber Li–S battery with different bending angles. (f) Rate performances of Ti_3_C_2_T*_x_*/nanoS-PVP@Al cathode at 0°C, 30°C and 60°C. (g) The capacity and specific capacity of fiber Li–S battery with different lengths. (h) Comparisons of the linear and specific capacity of this fiber Li–S battery with previously reported flexible fiber batteries.

The composite interface interaction and remarkable sulfur species encapsulation and adsorption ability make the Ti_3_C_2_T*_x_*/nanoS-PVP@Al an ideal cathode for forming flexible Li–S batteries. The electrochemical performance of Ti_3_C_2_T*_x_*/nanoS-PVP@Al, Ti_3_C_2_T*_x_*/S@Al and C/nanoS-PVP@Al as cathodes for fiber Li–S batteries was evaluated. CV curves of different cathodes with a scan rate of 0.1 mV s^−1^ are displayed in Fig. 4a. The Ti_3_C_2_T*_x_*/nanoS-PVP@Al cathode exhibits two pairs of redox peaks, corresponding to the serial oxidation/reduction of solid S_8_ to soluble long-chain Li_2_S*_x_* (4 ≤ *x* ≤ 8) and solid Li_2_S_2_/Li_2_S [34]. Compared with Ti_3_C_2_T*_x_*/S@Al and C/nanoS-PVP@Al, the Ti_3_C_2_T*_x_*/nanoS-PVP@Al cathode delivers the lowest voltage polarization and highest peak current density, revealing the increased capacity and enhanced reaction kinetics that are further confirmed by using galvanostatic charge/discharge plots (Fig. 4b) and electrochemical impedance spectroscopy (EIS) analysis (Fig. S12). As shown in Fig. 4b, the Ti_3_C_2_T*_x_*/nanoS-PVP cathode presents a polarization of ∼0.41 V at a constant current density of 0.1 C, which is much lower than those of the Ti_3_C_2_T*_x_*/S@Al and C/nanoS-PVP@Al counterparts, which are >0.45 V. EIS analysis of assembled cells shows a semicircle in the high-frequency range, which contains charge-transfer resistance (R_ct_) and overlapped solid–electrolyte interphase resistance (R_s_) [35]. The R_ct_ values of Ti_3_C_2_T*_x_*/nanoS-PVP@Al, Ti_3_C_2_T*_x_*/S@Al and C/nanoS-PVP@Al are approximately 64.2, 72.6 and 107.1 Ω, respectively, demonstrating the fastest electrochemical reaction kinetics of the Ti_3_C_2_T*_x_*/nanoS-PVP@Al cathode. The above results indicate that the Ti_3_C_2_T*_x_*/nanoS-PVP@Al cathode has enhanced electrochemical redox reaction kinetics, largely attributed to the hierarchical electrode structure and excellent catalytic conversion performance. First, few-layer Ti_3_C_2_T*_x_* links charge transport paths and provide space for the loading of nanoS, while composite PVP chains on nanoS particles prevent the agglomeration and detachment of nanoS via interface interactions such as H-bond forces. Second, soluble lithium polysulfides can be effectively limited to the cathode side due to multiscale physical barriers and plentiful chemisorption sites for polysulfides, thus decelerating the ‘shuttle effect’ during the reaction process. Accordingly, the Ti_3_C_2_T*_x_*/nanoS-PVP@Al cathode exhibits higher rate performance (Fig. 4c) and more stable cycling performance (Fig. 4d). The rate superiority is revealed with higher discharge capacities of 1210.9 (0.1 C), 990.2 (0.2 C), 812.8 (0.5 C), 716.8 (1.0 C), 556.2 (2.0 C) and 1054.2 (0.1 C) mAh g^−1^ being achieved, which are much better than those of the Ti_3_C_2_T*_x_*/S@Al and C/nanoS-PVP@Al cathodes. For long-term cycling performance, the Ti_3_C_2_T*_x_*/nanoS-PVP@Al cathode maintains a capacity of 912.6 mAh g^−1^ after 100 cycles at 0.1 C, with a capacity retention of 74.8%, which is superior to those of the Ti_3_C_2_T*_x_*/S@Al (549.2 mAh g^−1^, 45.0%) and C/nanoS-PVP@Al (434.5 mAh g^−1^, 36.8%, 50 cycles) cathodes. Furthermore, 99.1% of the overall coulombic efficiency of the Ti_3_C_2_T*_x_*/nanoS-PVP@Al cathode can be achieved during the cycling, which is higher than those of the Ti_3_C_2_T*_x_*/S@Al (98.7%) and C/nanoS-PVP@Al (96.6%) counterparts. Impressively, even at a high current rate of 1.0 C, the Ti_3_C_2_T*_x_*/nanoS-PVP@Al cathode preserves an exceptional cycling performance with an initial specific discharge capacity of 674.9 mAh g^−1^ and capacity retention of 92.8% after 1000 cycles (Fig. S13).

Fiber-shaped batteries may work under deformation and high/low-temperature adversity. The electrochemical performances of the fiber Li–S battery assembled with a Ti_3_C_2_T*_x_*/nanoS-PVP@Al cathode under different bending states and temperatures were investigated (Fig. 4e and f, and Fig. S14). The specific capacity of the fiber Li–S battery showcases almost negligible capacity degradation after bending from 0° to 60°, 90°, 120° and back to 0° (Fig. 4e), indicating excellent flexible energy-storage capability. Meanwhile, even after repetitive bending for 1000 cycles, the fiber battery retains ∼98.8% capacity (Fig. S14), demonstrating superior flexibility and durability, which are highly desired for wearable electronic devices. Furthermore, the high/low-temperature tolerance of the fiber Li–S battery is shown in Fig. 4f. Under conditions of 0°C and 60°C, the fiber battery operates normally at different current densities (0.1–2.0 C) and displays initial specific capacities of 1116.4 and 1024.7 mAh g^−1^ at 0.1 C, respectively, reaching 93.0% and 85.4% of the battery performance at 30°C (1200.4 mAh g^−1^). In addition to excellent gravimetric capacity (attributed to the cathode), the fiber battery achieved a high linear capacity, as shown in Fig. 4g. Within a certain range (1–10 cm), a linear relationship between the capacity and fiber length is proved, with a slope of the fitted line indicating a linear capacity of ∼22.9 mAh m^−1^. Compared with representative fiber batteries measured in specific and linear capacity, our prepared fiber Li–S battery exhibits great advantages (Fig. 4h), paving a promising path for next-generation wearable electronics [3,36–42].

### Applications of fiber Li–S batteries

**Figure 5.**
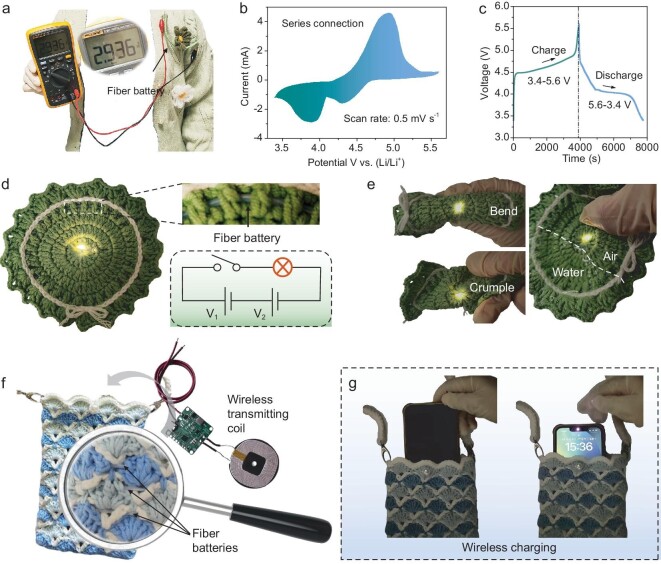
(a) Photograph of the output voltage of the fiber Li–S battery. (b) CV curve and (c) charge/discharge plots of two fiber batteries in series connection. Two fiber Li–S batteries were woven into a soft cloth (d) to light a yellow LED and (e) light an LED while bent, crumpled or submerged in water. (f) Schematic of the design strategy for wireless charging phone bag. (g) Photographs of a smartphone before and after wireless charging by the phone bag.

The open-circuit voltage of a single Li–S fiber battery is ∼2.94 V (Fig. 5a). When two fibers are assembled in series, the obtained output voltage doubles, as shown in the CV curve (Fig. 5b) and galvanostatic charge/discharge plots (Fig. 5c). Fiber Li–S batteries can be integrated into commercial textiles to power wearable devices. In this case, the fiber Li–S batteries were woven into a soft cloth to light up an LED. As shown in Fig. 5d and e, two fiber batteries in series easily light up a yellow LED and function normally even when bent, crumpled or submerged in water. Moreover, the fiber batteries were woven into a phone bag for wireless charging. As demonstrated in Fig. 5f, fiber Li–S batteries and a wireless transmitting coil were connected and embedded in a commercial phone bag. The charging process can be triggered when the smartphone is stuffed into the bag and reaches the wireless coil sensing area (Fig. 5g and Video S1). These results highlight the promising potential of fiber Li–S batteries for application in next-generation wearable electronic devices and smart fabrics.

## CONCLUSION

In summary, a highly flexible and mechanically robust composite fiber sulfur cathode with significant interface interactions was prepared by cascading assemblies of nanoS-PVP and few-layer Ti_3_C_2_T*_x_*. Notably, nanoS and polysulfides were synergistically hosted by Ti_3_C_2_T*_x_* with a synergistic effect of physical confinement and chemical adsorption. Meanwhile, the H-bond interaction between PVP chains wrapped in nanoS and Ti_3_C_2_T*_x_* prevented active sulfur from falling off during cathode deformation. The fabricated Ti_3_C_2_T*_x_*/nanoS-PVP@Al fiber cathode exhibited excellent cycling performance (capacity retention of 92.8% after 1000 cycles at 1 C), high-rate capacity (556.2 mAh g^−1^ at 2.0 C) and high linear capacity of ∼22.9 mAh m^−1^. Moreover, fiber Li–S batteries based on a Ti_3_C_2_T*_x_*/nanoS-PVP@Al fiber cathode showed sufficient flexibility and stability to endure 1000 deformation cycles and work effectively under high and low temperatures. The fiber batteries were integrated into commercial textiles to power LEDs or woven into a phone bag for wirelessly charging a smartphone. This work provides new insights into the preparation of high-performance wearable energy-storage devices.

## Supplementary Material

nwae262_Supplemental_Files
