# Supplementary material for: Interface-reinforced high-capacity fiber cathode for wearable Li–S batteries
Source: Natl Sci Rev. 2024 Jul 30;11(10):nwae262. doi: 10.1093/nsr/nwae262 (PMC11409864; doi:10.1093/nsr/nwae262)
Supplement: nwae262_Supplemental_Files [file nwae262_supplemental_files.zip › Supplementary data.pdf]

Supplemental Information

Interface-reinforced high-capacity fiber cathode for wearable Li-S batteries

Lei Huang[1], Tianzhu Zhou[1], Siyu Zhu[2], Tianqi Yang[3], Xuhui Zhou[1], Bing He[1], Shuai Wang[1], Wei Yan[4,*] and Lei Wei[1,*]

[1] School of Electrical and Electronic Engineering, Nanyang Technological University, Singapore 639798, Singapore;

[2] School of Materials Science and Engineering, Nanyang Technological University, Singapore 639798, Singapore;

[3] Department of Physics, City University of Hong Kong, Hong Kong 999077, China;

[4] State Key Laboratory for Modification of Chemical Fibers and Polymer Materials, College of Materials Science and Engineering, Donghua University, Shanghai 201620, China

* Corresponding author. E-mail: weiyan@dhu.edu.cn, wei.lei@ntu.edu.sg

Experimental Section

Preparation of nanoS-PVP

5 g sodium thiosulfate ($Na_2S_2O_3$) and 100 mg of polyvinylpyrrolidone K30 (PVP, MW: 40000) were dissolved in 500 mL deionized water and stirred for 30 min at 30 ℃. Subsequently, 20 mL hydrochloric acid (HCl, 0.5 mol L^{-1}) was dropwise added and continued stirring for 4 h. The nanoS-PVP particles were collected by centrifugation (8000 r min^{-1}) and washed several times with deionized water.

Preparation of few-layer $Ti_3C_2T_x$

Few-layer $Ti_3C_2T_x$ were synthesized by a modified method.[1] Specifically, 3.6 g pristine MAX (Ti_3AlC_2) powders and 7.6 g LiF were added to the HCl solution (80 mL, 9 M) and stirred at 50 ℃ for 30 h. The accordion-like precipitate was collected by centrifugation and washed with HCl and deionized water several times. The obtained nearly neutral sediments were dispersed into 200 mL deionized water with continuous vibration for 20 min and centrifuged at 1500 rpm for 30 min. Then the supernatant solution was centrifuged at 3500 rpm for 30 min to obtain sediments. The resulting sediments can be redispersed into deionized water to gain few-layer $Ti_3C_2T_X$ solutions with different concentrations.

Preparation of $Ti_3C_2T_x$/nanoS-PVP

100 mL few-layer $Ti_3C_2T_x$ solutions (5 g L^{-1}) were mixed with 4.5 g collected nanoS-PVP and stirred for 5 h. The mixed solution was collected by centrifugation and washed with deionized water several times. $Ti_3C_2T_x$/nanoS-PVP powders can be obtained with freeze drying for 48 h.

Preparation of fiber $Ti_3C_2T_x$/nanoS-PVP@Al cathode

The $Ti_3C_2T_x$/nanoS-PVP@Al cathode was fabricated by coating the slurry onto an Al fiber and dried at 60 ℃ for 12 h in a vacuum. The slurry was prepared by mixing $Ti_3C_2T_x$/nanoS-PVP with conductive carbon black and polyvinylidene fluoride (PVDF) at the weight ratio of 18:1:1 in N-methyl-2-pyrrolidone (NMP).

Preparation of gel electrolyte

0.5 g polyvinylidene fluoride‐hexafluoropropylene (PVDF-HFP) was dissolved in 10 mL liquid electrolyte at 80 ℃. The gel electrolyte was produced when the mixture

cooled down to room temperature. The liquid electrolyte was 1 M bis (trifluoromethane) sulfonamide lithium salt (LiTFSI) in a mixed solvent of 1,3-dioxolane (DOL) and 1,2-dimethoxyethane (DME) with a volume ratio of 1:1, including 2 wt. % $LiNO_3$ as an electrolyte additive. The electrolyte used in the cell was 25 μL mg^{-1}.

Preparation of Cu/Li fiber anode

The Cu/Li fiber anode was fabricated by a handmade platform in the glovebox filled with Ar. The Li metal was placed on a heating table at 300 ℃ until molten. The Cu fiber treated with air plasma was passed through the molten Li at a specific rate to obtain a Cu/Li composite fiber anode.

Materials and Characterization

All the chemical reagents are purchased from commercial sources and used without further purification. X-ray diffraction (XRD) characterizations were observed using XRD Shimadzu Powder equipment with a Cu Kα source (40 kV 30 mA, and 0.154 nm wavelength). The morphological characteristics of different samples were carried out by field-emission scanning electron microscopy (SEM, JEOL JSM-7600F) and transmission electron microscopy (TEM, JEOL 2100F-A). XPS spectrum was recorded using a Kratos AXIS Supra spectrometer. The mechanical measurement of fiber was tested using a tensile testing machine (KJ-1065), with a tensile rate of 2 mm min^{-1} and a standard length of 30 mm. Thermogravimetric (TG) analysis was carried out using a Netzsch STA 449C thermal analyzer. Fourier transform infrared spectra (FTIR) were recorded on the NICOLET iS50 FT-IR spectrometer. The *ex-situ* visible adsorption measurements were performed using $Ti_3C_2T_X$ and carbon with equal mass to soak into 0.5 mmol L^{-1} Li_2S_6 in DME: DOL=1:1 vol.%. UV-vis spectra of samples were gained from the Shimadzu UV-3600plus spectrophotometer.

Electrochemical characterization

The fiber Li-S batteries were assembled in a glovebox filled with Ar. The discharge/charge performances were tested on a LAND battery program-control test system (Wuhan, China) in a potential range between 1.7 and 2.8 V at room temperature, 0 ℃, 30 ℃, and 60 ℃. Electrochemical impedance spectroscopy (EIS)

measurements and Cyclic voltammetry (CV) curves were performed on an electrochemical workstation (CHI 760E, Chenhua). The specific capacity was calculated based on the mass of sulfur in the electrode.

Computational details:

DFT Parameters. The spin-polarized DFT calculations were carried out using the Vienna ab initio simulation package (VASP). The exchange-correlation potential was treated by the Perdew-Burke-Ernzerhof (PBE) of generalized gradient approximation (GGA) and the long-range van der Waals interaction between adsorbates and surfaces was considered by Grimme's zero-damped DFT-D3 method. The projector augmented wave method (PAW) with a plane-wave kinetic energy cutoff of 400 eV was used to expand the valence states of all atoms. The Brillouin zone integration was sampled using the Monkhorst-Pack scheme, and the meshes used for $Ti_3C_2T_x$-OH and carbon surfaces were $3 \times 3 \times 1$. All the geometry optimization would be converged when the energy difference was smaller than 5.0×10^{-6} eV, and the forces were less than 0.02 eV/Å.

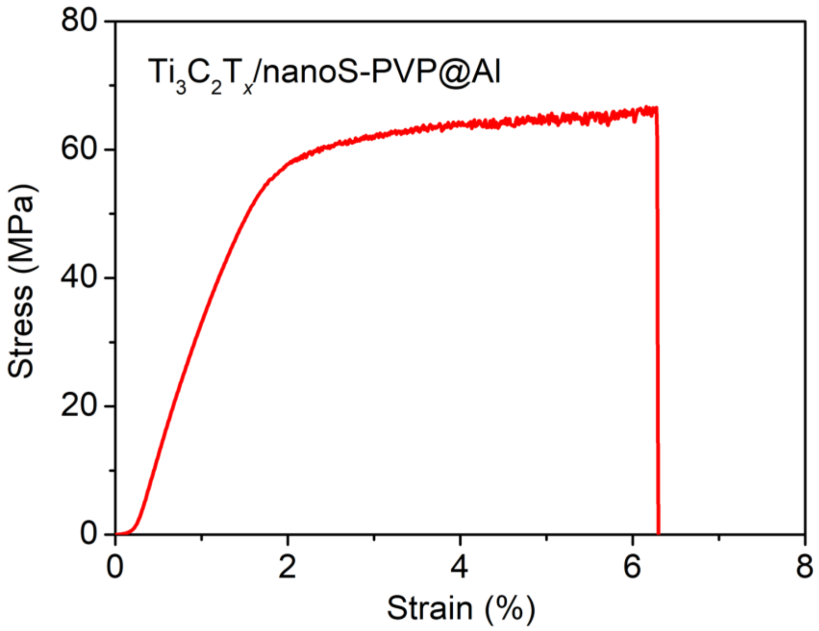

Figure S1. Stress-strain curve of fiber $Ti_3C_2T_x$/nanoS-PVP@Al cathode.

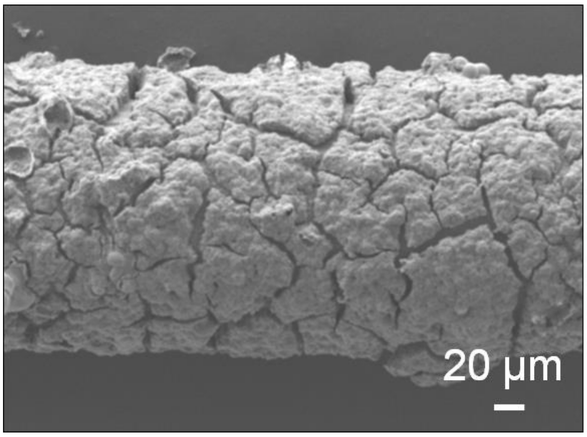

Figure S2. SEM image of carbon/nanoS-PVP@Al fiber cathode.

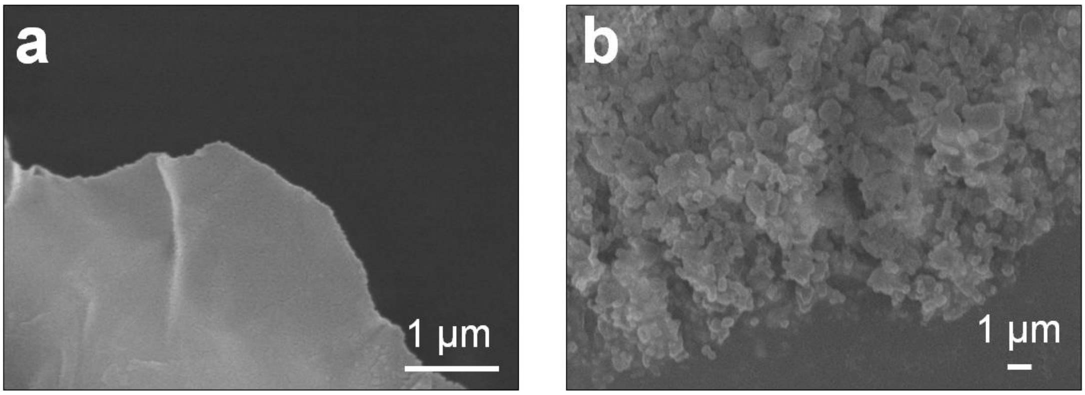

Figure S3. SEM images of (a) few-layer $Ti_3C_2T_x$ and (b) nanoS-PVP.

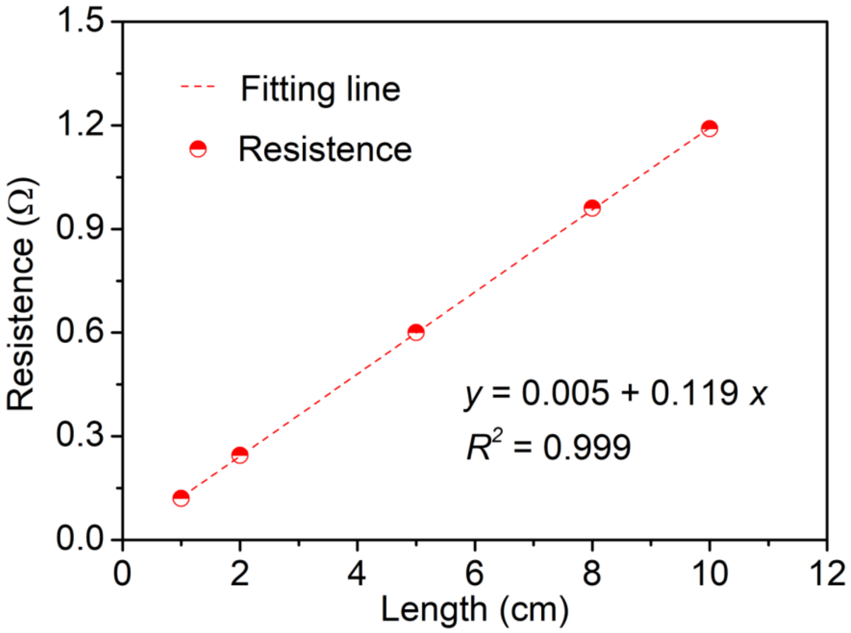

Figure S4. Length-resistance curves of $Ti_3C_2T_x$/nanoS-PVP@Al cathode.

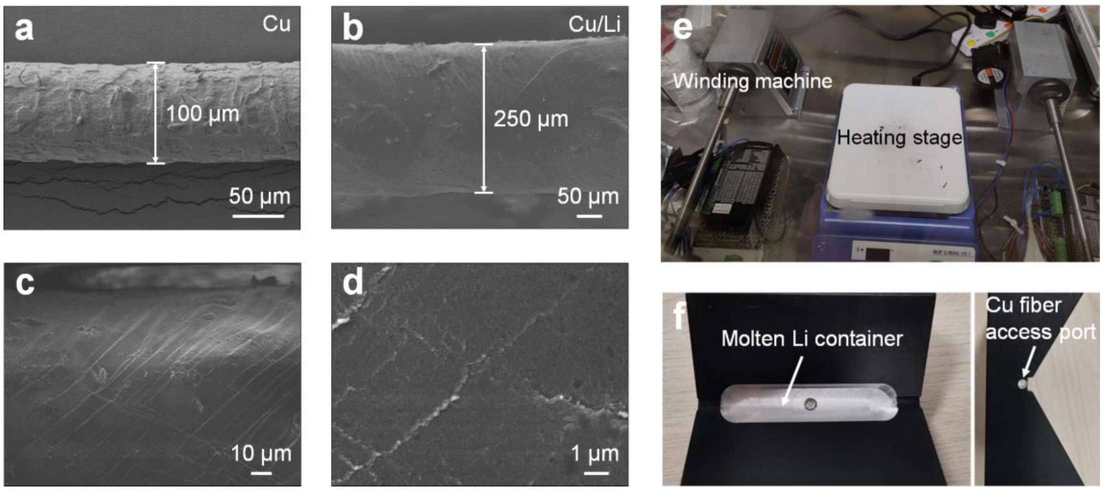

Figure S5. SEM images of (a) Cu fiber and (b-d) Cu/Li fiber anode. (e-f) The handmade platform for Cu/Li fiber anode preparation.

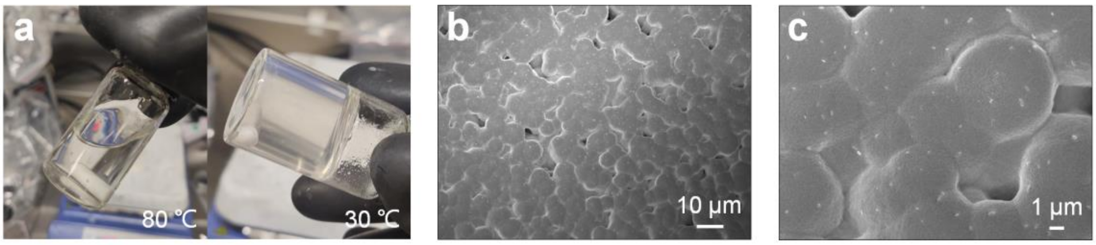

Figure S6. (a) Photographs of gel electrolyte at 30 ℃ and 80 ℃. (b-c) SEM images of gel electrolyte after drying.

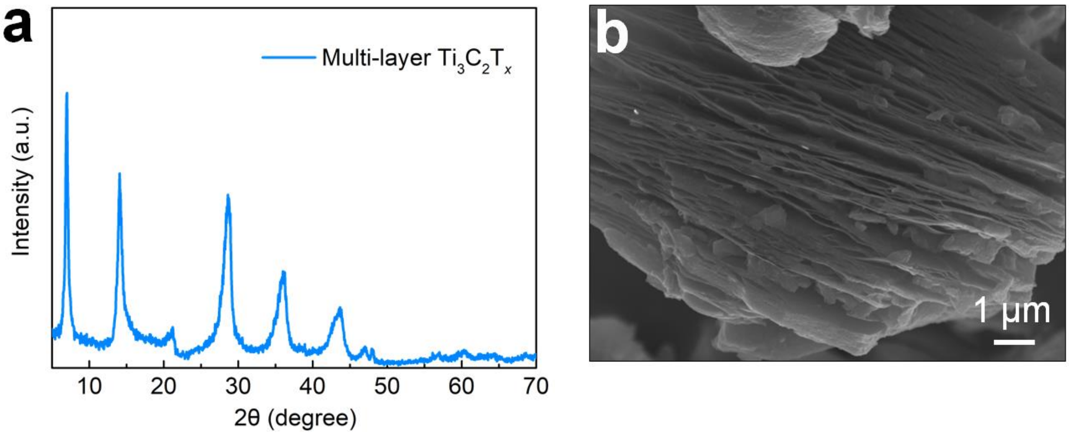

Figure S7. (a) XRD pattern and (b) SEM image of multi-layer $Ti_3C_2T_x$.

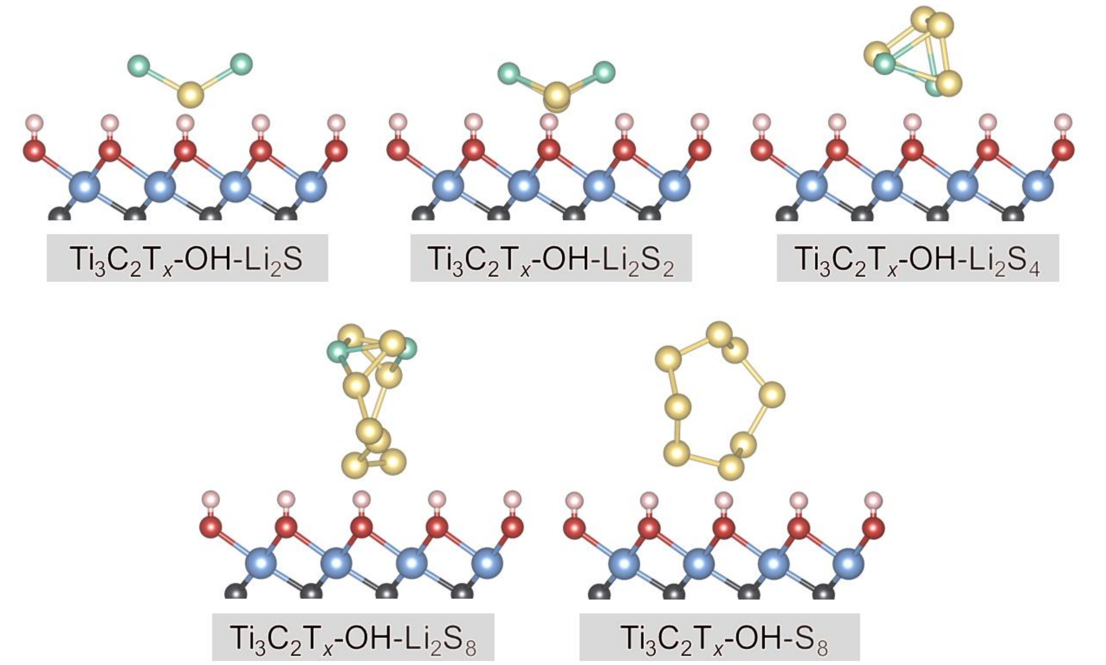

Figure S8. Front views of sulfur species (Li_2S, Li_2S_2, Li_2S_4, Li_2S_8, and S_8) adsorbed on $Ti_3C_2T_x$-OH.

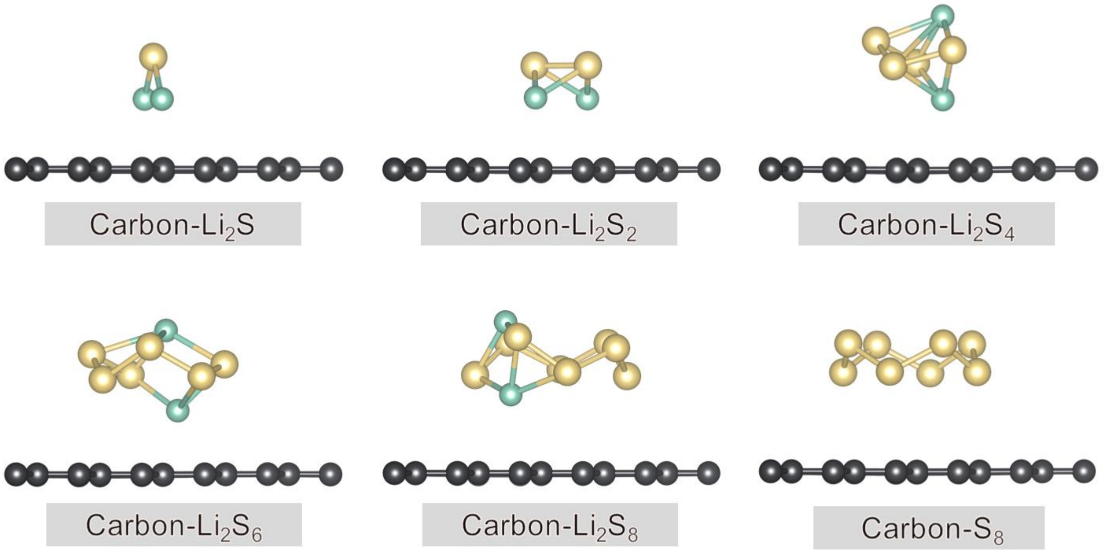

Figure S9. Front views of sulfur species (Li_2S, Li_2S_2, Li_2S_4, Li_2S_6, Li_2S_8, and S_8) adsorbed on carbon.

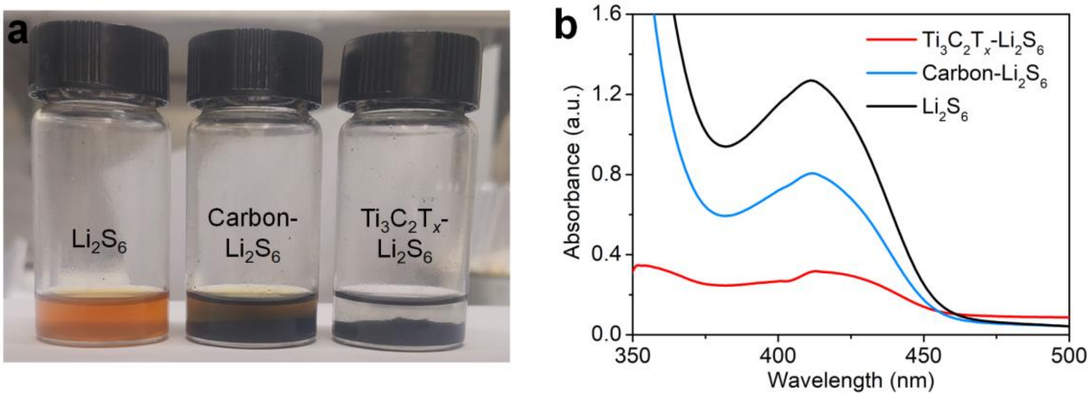

Figure S10. Adsorption test for Li_2S_6 on $Ti_3C_2T_x$ and carbon samples. (a) Optical photos of Li_2S_6 solution adsorbed by $Ti_3C_2T_x$ and carbon at 24 h. (b) UV-vis spectra of Li_2S_6 solution, Li_2S_6 solution with $Ti_3C_2T_x$, and carbon samples.

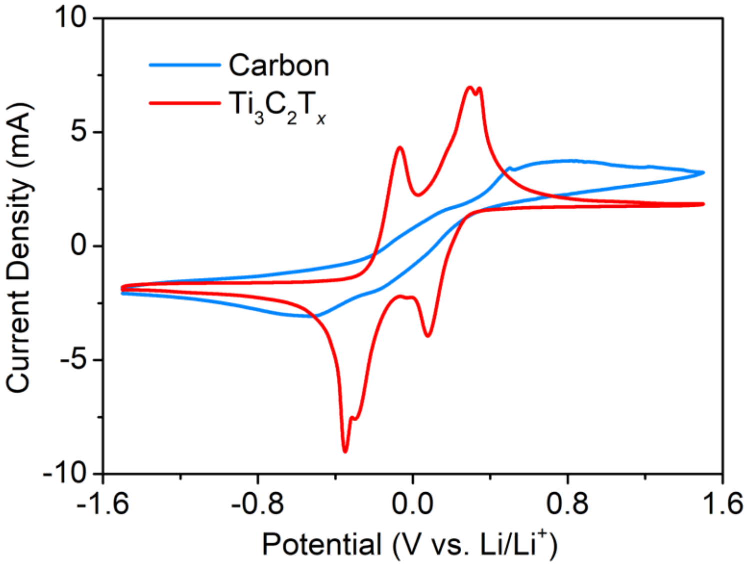

Figure S11. CV curves of Li_2S_6 symmetric cells at a scan rate of 10 mV s^{-1}.

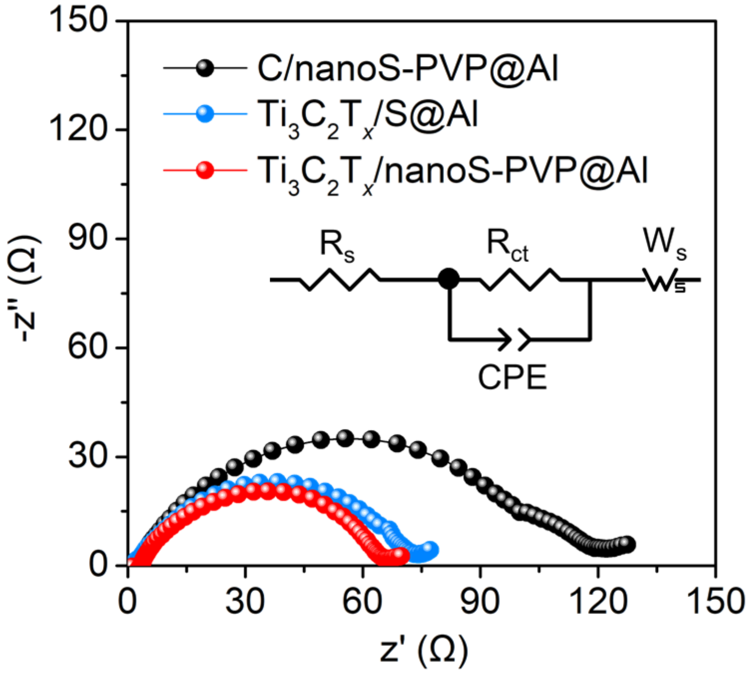

Figure S12. Nyquist plots of $Ti_3C_2T_x$/nanoS-PVP@Al, $Ti_3C_2T_x$/S@Al, and C/nanoS-PVP@Al fiber cathodes.

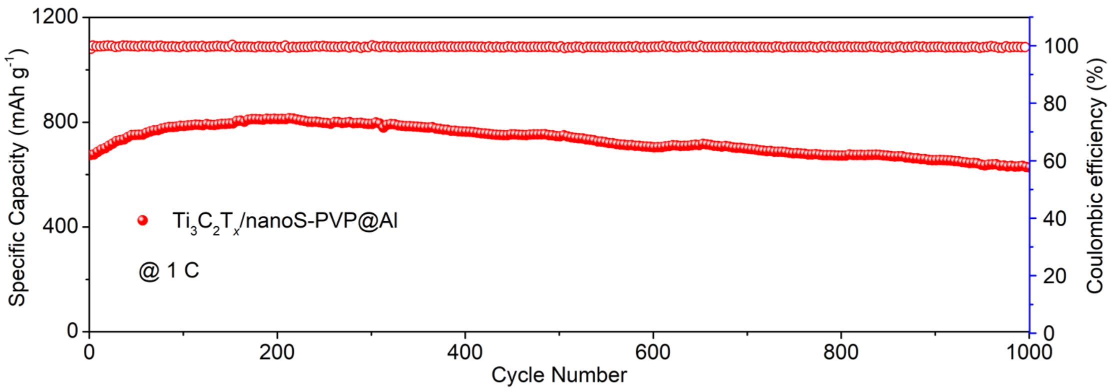

Figure S13. Cycling performance of $Ti_3C_2T_x$/nanoS-PVP@Al fiber cathode at 1 C.

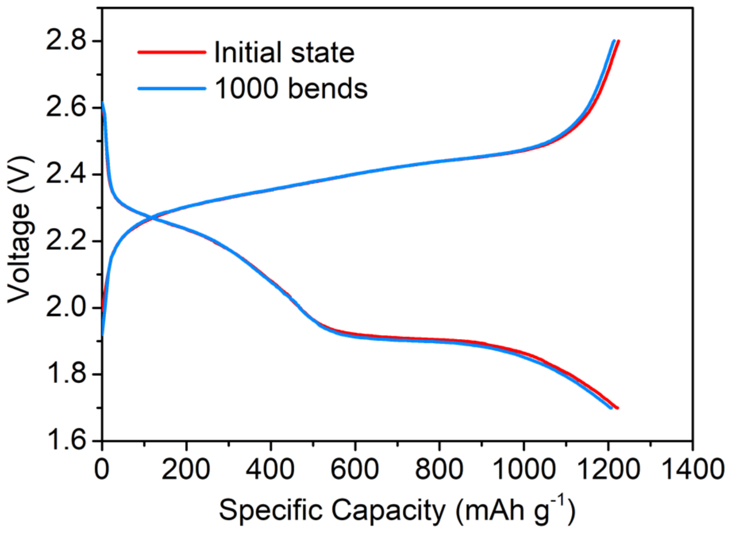

Figure S14. Galvanostatic charge/discharge plots of $Ti_3C_2T_x$/nanoS-PVP@Al fiber cathode before and after repetitive bending for 1000 cycles.

References

1. Zhou T, Cao C, Yuan S *et al.* Interlocking-governed ultra-strong and highly conductive MXene fibers through fluidics-assisted thermal drawing. *Adv Mater* 2023; **35**: 2305807.
